# Poster Session II - A314 IMPLEMENTATION OF ACCEPTANCE AND COMMITMENT THERAPY IN INFLAMMATORY BOWEL DISEASE: A SCOPING REVIEW

**DOI:** 10.1093/jcag/gwaf042.314

**Published:** 2026-02-13

**Authors:** C Long, N Willett, K Kidd, E Jones, S Farina, J Jones

**Affiliations:** Dalhousie University, Halifax, NS, Canada; Dalhousie University, Halifax, NS, Canada; Dalhousie University, Halifax, NS, Canada; Dalhousie University, Halifax, NS, Canada; Dalhousie University, Halifax, NS, Canada; Dalhousie University, Halifax, NS, Canada

## Abstract

**Background:**

Psychological distress is common in patients with inflammatory bowel disease (IBD) and worsens symptoms and disease activity. Acceptance and commitment therapy (ACT) has been shown to be beneficial in chronic disease-related psychological distress. Although access to mental health resources improves clinical and quality of life outcomes, IBD patients face barriers in the accessability and, once accessed, persistence in these therapies.

**Aims:**

The aim is to describe the implementation effectiveness of ACT interventions for psychological distress in IBD patients.

**Methods:**

This scoping review followed the preferred reporting items for systematic reviews and meta-analyses (PRISMA) guidelines. The databases CINAHL, Cochrane Library, Embase, OVID, MEDLINE, and PsychInfo were systematically searched. Two authors independently screened and extracted data from the articles.

**Results:**

The literature review identified 49 unique sources. Thirteen studies conducted from 2015-2024 were included for analysis, with 3 randomized controlled trials, 3 observational, 2 qualitative, 1 quasi experimental, 1 protocol corrigendum, and 1 series of single cases. Sample size ranged from 5 to 122 subjects. There were 10 unique interventions, most of which were 8 weeklong courses. Interventions were online (n = 2), in person (n = 2), or unspecified (n = 6). Three were group-based ranging from 6 to 16 participants. Most included workbooks or homework. Five were therapist-led.

Seven studies reported effectiveness data with overall improvement in psychological outcomes. There was variability in sustained reduction of anxiety and depression scores. Two studies explicitly reported on feasibility with variation of attrition rates. Focus group data suggest that programs should account for disease specific factors such as timing in the illness trajectory (i.e. at diagnosis and after relapse) and patient factors including literacy, motivation, psychological comorbidities, and lifestyle. Participants preferred a virtual platform or written material to improve accessibility, but there was ambivalence around online self-directed modules.

**Conclusions:**

This review highlights the diversity of ACT treatments for IBD patients, with 10 unique interventions included. Overall, ACT is effective in reducing anxiety and acceptable to patients, however effect sizes varied with inconsistent sustained response on follow up likely due to differences in programming, implementation strategy, patient baseline characteristics, and therapeutic persistence. Future research should address barriers and facilitators for ACT interventions to improve accessibility, acceptability, and feasibility for IBD patients.

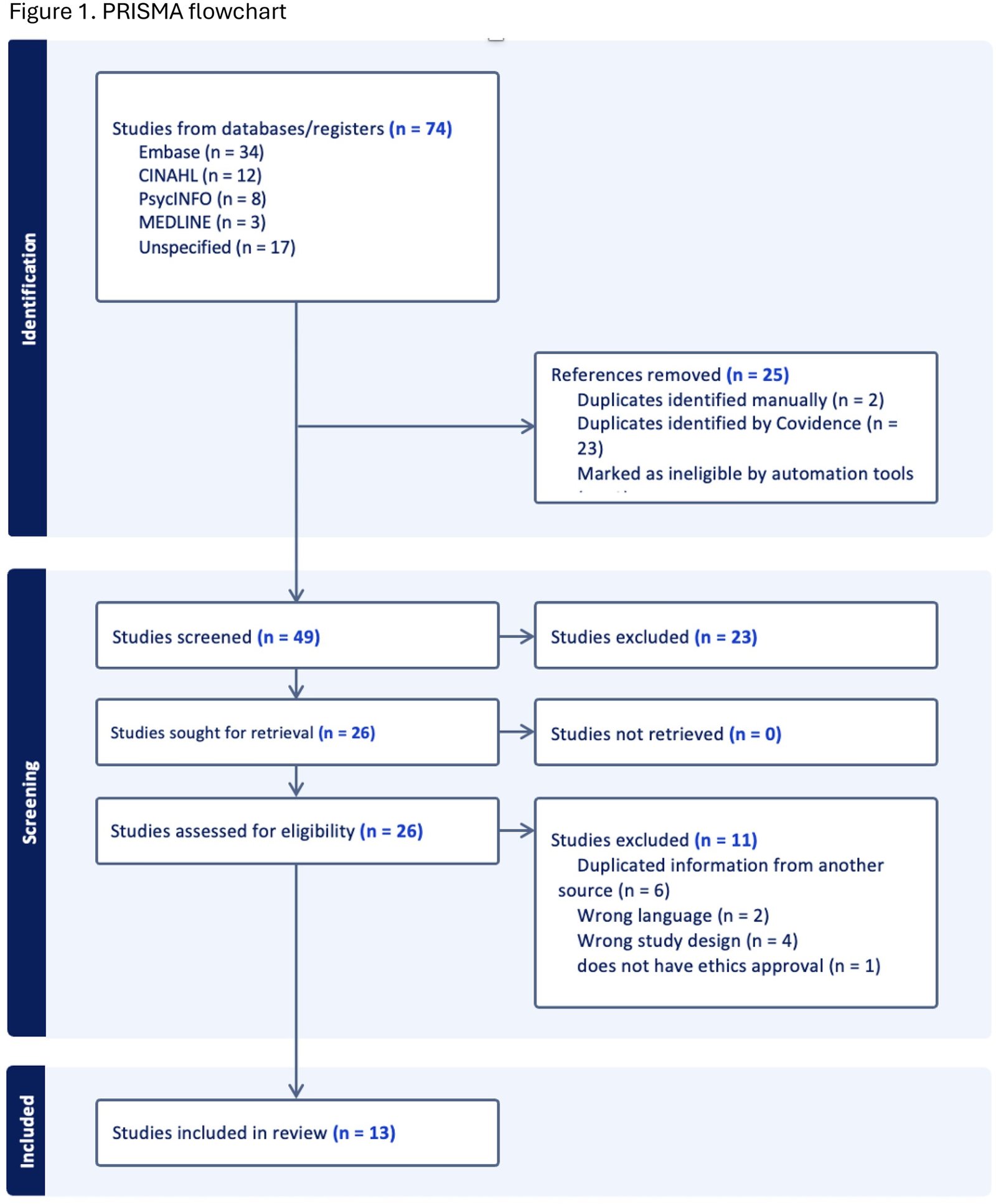

**Funding Agencies:**

None

